# Serious Games Supporting the Prevention and Treatment of Alcohol and Drug Consumption in Youth: Scoping Review

**DOI:** 10.2196/39086

**Published:** 2022-08-25

**Authors:** Juan Martínez-Miranda, Ismael Edrein Espinosa-Curiel

**Affiliations:** 1 Centro de Investigación Científica y de Educación Superior de Ensenada Unidad de Transferencia Tecnológica Tepic Tepic Mexico

**Keywords:** serious games, substance use, alcohol and drugs, young population, mobile phone

## Abstract

**Background:**

The consumption of alcohol and drugs, particularly in adolescents and young adults, has increased worldwide in the last several years, representing a significant public health challenge. Serious games have the potential to support preventive and treatment interventions for substance use, facilitating the acquisition of relevant knowledge and the motivation for changes in attitudes and behaviors regarding substance consumption.

**Objective:**

This scoping review aims to analyze a set of 7 relevant characteristics of current serious games designed to support the prevention and treatment of alcohol and drug consumption in adolescents and young adults—the substance addressed, the type of intervention, the theoretical basis, the computational techniques used, the mechanism for data security and privacy, the evaluation procedure followed, and the main results obtained.

**Methods:**

The review was performed by following the PRISMA-ScR (Preferred Reporting Items for Systematic Reviews and Meta-Analyses extension for Scoping Reviews) guidelines. Data were retrieved from January 2010 to May 2022, using PubMed, Scopus (Elsevier), IEEE Xplore, and ACM Digital as data sources. The eligibility criteria included studies that described serious games designed to support the prevention or treatment of alcohol and drug consumption, targeted a population aged between 12 and 30 years, and included an evaluation procedure. Authors (JMM and IEEC) individually screened the titles and abstracts, and then full articles were reviewed for a final inclusion decision.

**Results:**

A total of 629 records were obtained, and 29 (4.6%) fulfilled the inclusion criteria. Most of the serious games (14/29, 48%) were focused on the prevention or treatment of alcohol use. The type of intervention that was the most supported was prevention (18/29, 62%), and most studies mentioned the theory, theoretical construct, or therapeutic technique used as a foundation (22/29, 76%). Most of the studies only provided information about the platform for execution (23/29, 79%), and few described the use of computational techniques, such as virtual reality or motion-based interaction (5/29, 17%). A small set of studies (10/29, 34%) explicitly mentioned how data security and privacy were addressed. Most of the reported evaluation protocols were pilot studies (11/29, 38%), followed by randomized controlled trials (10/29, 34%), and the reported results were positive in terms of acceptability, usability, and efficacy. However, more research is needed to assess long-term effects.

**Conclusions:**

Given the increasing interest in the use of serious games as digital interventions to support the prevention or treatment of substance use, knowing their main features is highly important. This review highlights whether and how current serious games incorporate 7 key features that are useful to consider for the further development of the area.

## Introduction

### Background

The use of illicit drugs and alcohol is a serious public health problem worldwide that affects an important percentage of the population. The United Nations World Drug Report 2021 estimates that approximately 5.5% of the population aged between 15 and 64 years have used drugs at least once in the past year, whereas 36.3 million people (13% of the total number of people who use drugs) have drug use disorders [1]. Furthermore, the World Health Organization (WHO) estimates that the total alcohol consumption per capita in the world’s population aged ≥15 years is at the level of 6.4 L of pure alcohol, and it is expected to increase to 7.0 L in 2025 [2].

Adolescence is the developmental period with the highest risk of developing a substance use disorder [3]. Studies on brain development indicate that this period of life is characterized by suboptimal decisions and actions that are associated with risk behaviors, including an increased incidence of substance abuse [4]. The health consequences of alcohol and drugs in this population are highly negative as the consumption of substances is associated with neurocognitive alterations that can lead to behavioral, emotional, social, and academic problems later in life [5]. Moreover, some studies show that people of younger ages are disproportionately affected by alcohol when compared with individuals of older ages. The proportion of all deaths attributable to alcohol consumption is most significant among those aged 20 to 39 years, representing 13.5% of all deaths among these people [2]. Furthermore, drug overdose is behind the deaths of many adolescents, which have increased in the last decades, mainly because of the consumption of opioids [6]. Thus, interventions for preventing and treating alcohol and drug consumption in youth are necessary to tackle substance abuse to minimize the negative consequences in older ages. These interventions are crucial during adolescence and emerging adulthood because of evidence that precocity has been associated with a further increased risk of developing substance use disorders [7].

Different intervention strategies are currently implemented to mitigate substance use in adolescents and young adults, including universal prevention, selective prevention, and treatment [8]. Although the evidence shows that these interventions are effective, there is also a critical need to increase the availability and accessibility of these health services to a greater population [9]. Owing to the wide use and acceptance of technology among the young population, different web-based applications and mobile apps have been developed to support and complement traditional interventions or are even provided as stand-alone interventions. These digital applications offer some advantages such as minimal cost [10], reducing the burden on health care professionals [11], and the personalization of the intervention to different individuals [12]. In addition to web-based applications and mobile apps, one of the digital solutions that would offer different advantages to support substance use interventions, particularly for adolescents and young adults, is the development of serious games.

Serious games are computer games developed with objectives not only related to users’ entertainment but also to facilitate the increase of skills and abilities, gain knowledge, or acquire experience. The use of serious games for health has increased in the last several years because of their capabilities to simulate real-life situations, collect information that helps identify specific conditions and behaviors, and provide information to involve and support the user at different stages of the health care process [13]. Serious games have been used as digital-based interventions for different health care problems such as the prevention of obesity in children [14], supporting children with chronic diseases [15], promoting the treatment of mental illness [16], and providing information about how to prevent COVID-19 [17], to name a few.

Substance use prevention and treatment are also benefiting from serious games. Different research efforts are currently dedicated to developing and evaluating serious games to facilitate the gain of knowledge and the promotion of behavior change in individuals facing health problems related to substance use. Thus, it is essential to know how these solutions are currently designed, implemented, and evaluated in terms of the following questions: (1) What are the substances whose use is intended to be prevented or treated with the support of serious games? (2) How are these serious games designed and used (as stand-alone interventions or as part of a prevention or treatment program)? (3) What is the theoretical background on which these serious games are based? (4) What are the main computer-based techniques and methods (eg, augmented reality, artificial intelligence, and brain-computer interfaces) implemented in these serious games? (5) What are the types of evaluation and what are the main outcomes assessed with these serious games? (6) What mechanism is implemented to assure the security and confidentiality of users’ data? (7) What are the reported efficacy and limitations of the serious games used for the prevention or treatment of substance use?

### Objectives

To our knowledge, there are 2 previous reviews addressing the use of serious games for the prevention of alcohol and drug use, but both are exclusively focused on educational purposes [18,19]. Thus, the objective of this study was to perform a scoping review of serious games used for the prevention and treatment of alcohol and other drug use considering not only educational or learning objectives but also the promotion of behavior change. By answering the aforementioned 7 questions, this review will contribute to a better understanding of how serious games are currently designed, used, and evaluated, as well as of the reported impact they have as digital interventions for the prevention and treatment of substance use in adolescents and young adults.

## Methods

We followed a scoping review methodology to synthesize concepts and research concerning the use of serious games as interventions designed to support the prevention and treatment of alcohol and drug consumption in youth. This protocol followed the PRISMA-ScR (Preferred Reporting Items for Systematic Reviews and Meta-Analyses extension for Scoping Reviews) methodology [20] to ensure that our review was conducted systematically and was bias-free.

### Eligibility Criteria

The studies included in this scoping review were studies published between January 2010 and May 2022 that focused on the description of (1) *serious video games* developed with the objective to (2) *support the prevention or treatment* of (3) *alcohol and drug use*, targeted (4) *a population aged between 12 and 30 years*, and included (5) an *evaluation procedure.*

#### Serious Games

To include only serious video games and exclude other types of interactive game-like applications (eg, applications implementing simulated virtual environments or conversational synthetic characters), the main inclusion criterion was to identify in the retrieved works the five components of a serious game described in the study by Wattanasoontorn et al [13]: (1) implements rule or gameplay, (2) contains a challenge, (3) implements some type of user interaction, (4) has an explicit objective (entertainment), and (5) has an implicit objective (increasing skills and abilities, gaining knowledge, or acquiring experience).

#### Drugs

When referring to drugs in this study, we adopt the WHO definition, as follows: psychoactive drugs that, when taken in or administered into one’s system, affect mental processes (eg, perception, consciousness, cognition, or mood and emotions). Psychoactive drugs belong to a broader category of psychoactive substances that also include alcohol and nicotine [21]. As we are also interested in substances that, if not identified and treated adequately, would cause mental health problems, in this review we excluded studies focused only on the prevention or treatment of tobacco use.

#### Adolescents and Youth Population

Owing to the evidence that adolescents and young adults are particularly affected by alcohol and drug consumption, we focused this review on serious games designed for and evaluated in this population. Nevertheless, there are no clear guidelines for determining what ages should be included in the designation of young adulthood. The WHO categorizes young people as adolescents and young adults from 10 through 24 years of age, and the United Nations defines youth as 15 to 24 years of age [22]. We decided to include all the studies considering the range from 12 to 30 years in line with other research that consider this age range when referring to adolescents and young adults in studies related to alcohol and drug consumption [23-25].

Thus, articles were excluded if they described studies (1) that did not describe a serious game or were not related to substance use, (2) whose main objective was not the prevention or treatment of substance use, (3) that did not include adolescents and young adults as the target population (age: range 12-30 years), (4) that did not include an evaluation procedure, (5) that described literature reviews, (6) that were focused only on the prevention or treatment of tobacco use, (7) whose main aim was the identification or screening of individuals at risk of substance use, (8) that were not in English, (9) that were repeated, and (10) that were not research articles.

### Information Sources

PubMed, Scopus (Elsevier), IEEE Xplore, and ACM Digital were used to search for published papers. These 4 databases cover medical and computer science literature, allowing for comprehensive topic and field searches. The first search concluded in October 2021, and the final search concluded in May 2022. Some papers cited in the retrieved articles (hand searched) were also considered to complete the search.

### Search

Depending on each database, the specific syntax of the queries was different but, in all cases, the same words were used to represent the constructs of (1) serious games together with (2) substance use but excluding (3) game and internet addiction. The query used was integrated with the following words: (“serious games” OR “gamification” OR “gamified” OR “games for health” OR “educational game” OR “videogame*” OR “game-based” OR “video game*”) AND (“substance use” OR “substance abuse” OR “substance addiction” OR “alcohol addiction” OR “alcohol use” OR “alcohol abuse” OR “drug use” OR “drug abuse” OR “drug addiction”) AND NOT (“game addiction” OR “gaming disorder” OR “gambling” OR “gaming problems” OR “smartphone addiction” OR “internet addiction” OR “video gaming”).

### Study Selection

A multistage screening process was conducted. First, 2 reviewers (JMM and IEEC) performed title and abstract screening. Articles that both researchers included then entered a second phase, where full texts were reviewed for the final inclusion decision. When some papers were related to the same study or application, the most recent one was selected unless significant differences were reported in the evaluation protocol (eg, the inclusion of different outcomes or testing with a different target population). The review of the articles and the data extraction were carried out separately, and any disagreements were resolved through discussion until a consensus was reached.

### Data Charting and Synthesis of Results

All the studies meeting the inclusion criteria were selected for the review, and the data extracted were those that allowed for the answering of the seven questions listed in the Introduction section: (1) the type of substance use (alcohol or other drugs) the serious game aimed to prevent or treat, (2) the type of intervention that the serious game supported (as a stand-alone application or as part of a prevention or treatment program), (3) the theoretical basis used as the background model of the serious game, (4) the main computer-based methods or techniques implemented in the serious game, (5) the type of evaluation and procedure, (6) the mechanism implemented to maintain the security or confidentiality of users’ data, and (7) the reported results in terms of the different outcomes relevant to the prevention or treatment of substance use (eg, gain of knowledge, skill development, or behavior change).

The authors developed, calibrated, and used a template containing different sections to extract and summarize the aforementioned data. We further describe the main findings that emerged across the studies.

## Results

### Overview

**Figure 1:**
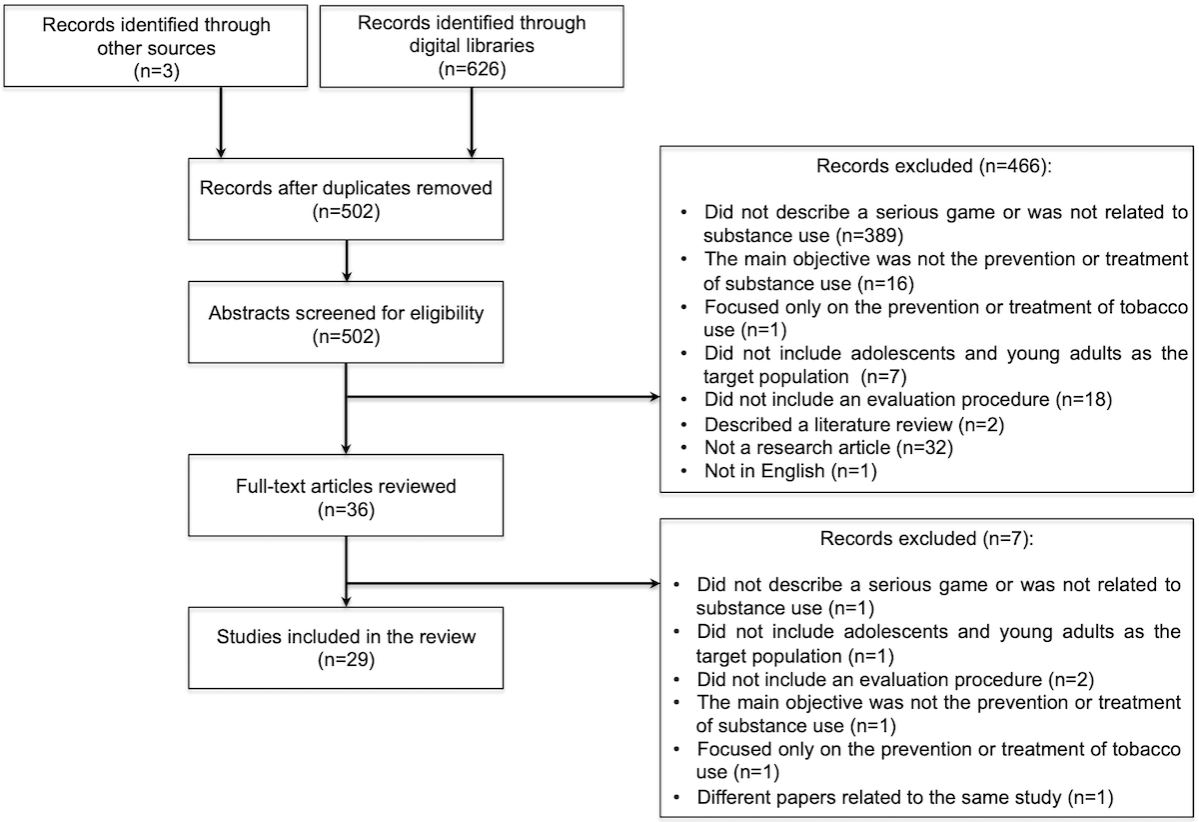
Flow diagram of the review phases indicating the reasons for exclusion.

A total of 629 records were identified from the searching process. From the 4 digital libraries, 99.5% (626/629) of the records were retrieved, and an additional 0.5% (3/629) of papers were obtained through hand searching. The records retrieved from each digital library were as follows: 21.1% (133/629) from PubMed, 33.1% (208/629) from Scopus, 26.4% (166/629) from IEEE Xplore, and 18.9% (119/629) from ACM Digital. In the first stage, after removing all the duplicated records, a total of 502 papers were screened for eligibility. After reading the titles and abstracts, 92.8% (466/502) of the records were discarded based on the exclusion criteria. A total of 36 articles were full-text reviewed, and 7 (19%) were excluded after the review. As a result, 29 studies were considered for further analysis. Figure 1 presents the flow diagram of the different review phases.

Most of the 29 studies were conducted in North America (United States and Canada; n=16, 55%), followed by studies developed in Europe (n=6, 21%), Australia (n=3, 10%), Brazil (n=2, 7%), and the Philippines (n=2, 7%). Most of the reviewed serious games (14/29, 48%) were developed to address problems associated with the consumption of alcohol. The type of intervention mainly supported was prevention (18/29, 62%), and the type of evaluation protocol most reported was pilot study (11/29, 38%) followed by randomized controlled trial (RCT; 10/29, 34%). In terms of the computational techniques used, most of the reviewed serious games only reported the type of platform where the game could be executed: web-based (11/29, 38%), mobile-based (7/29, 24%), multi-platform (3/29, 10%), or PC (2/29, 7%). Table 1 presents details of the general results. The following subsections describe the main findings to answer the 7 defined questions in detail.

**Table 1:** Overview of the general results related to some of the assessed characteristics of the serious games (N=29).

Characteristic			Studies, n (%)
**Type of substance addressed**			
	Alcohol	14 (48)	
	Drugs	8 (28)	
	Alcohol and drugs	7 (24)	
**Type of intervention**			
	Supporting prevention	18 (62)	
	Supporting treatment	11 (38)	
**Evaluation protocol**			
	Pilot study	11 (38)	
	RCT^a^	10 (34)	
	Quasi-experimental study	5 (17)	
	Comparative study	2 (7)	
	Proof-of-concept study	1 (3)	
**Computational techniques used**			
	Web-based	11 (38)	
	Mobile-based	7 (24)	
	Multi-platform	3 (10)	
	Use of virtual reality	3 (10)	
	Use of Kinect	2 (7)	
	PC-based	2 (7)	
	Not specified	1 (3)	

^a^RCT: randomized controlled trial.

### Type of Substance Addressed

In terms of the substances addressed, of the 29 studies, 13 (45%) were focused on problems associated with the consumption or abuse of alcohol, 7 (24%) were focused on drugs (such as methamphetamine, inhalants, cannabis, ecstasy, opioids, lysergic acid diethylamide, cocaine, and heroin), and 7 (24%) addressed problems for both alcohol and drug use. It is important to note that 7% (2/29) of the studies considered comorbidity—of these 2 studies, 1 (50%) focused on alcohol use disorder and depression [26,27], and 1 (50%) focused on substance use and relationship violence [28].

### Type of Intervention

Most of the evaluated serious games (18/29, 62%) were developed to support the prevention of substance use, whereas the rest of the serious games (11/29, 38%) were focused on the enhancement of the provided treatment or on supporting individuals during specific stages of their treatment. All the serious games designed with prevention objectives (18/29, 62%) implemented their games’ mechanics to facilitate the increase of knowledge on different topics, such as the negative consequences of nonmedical prescription drug use [29], the biological consequences of the abuse of inhalants [30], how the brain is affected by alcohol and other drugs [31,32], how to identify and reduce risky health behaviors [33,34] as well as carry out protective behavioral techniques [35,36], and the effects and consequences of alcohol and drug consumption [37,38].

Complementarily, the serious games focused on treatment (11/29, 38%) were designed to complement the therapeutic process for individuals with addiction problems or at risk and minimize relapse [39,40], reduce consumption [41-44], support recovery efforts [45], and promote a better adherence to the treatment [26,27]. Regarding the design of use, most of the serious games developed with prevention objectives (18/29, 62%) were stand-alone applications, and just a few (4/13, 31%) were designed as part of school-based prevention courses or curricula where teachers or social workers acted as facilitators [28,29,32,36,46]. On the contrary, most of the serious games developed with objectives to support treatment (11/29, 38%) were designed as technological-based tools to be used under the supervision of treatment providers.

### Theoretical Background

The theoretical backgrounds used as the foundation of the analyzed serious games were varied in those studies that explicitly mentioned their theoretical basis (22/29, 76%). A total of 14 studies (14/29, 48%) referred either to a specific theory or theories, a theoretical construct, or an intervention technique. Only a few studies (8/29, 28%) referred to a theory jointly with the particular theoretical constructs or intervention techniques used in the serious game [33,40-44,47,48]. Many of the features designed in the serious games for knowledge increasing and the fostering of health behavior adoption were based on the theory of reasoned action [29,47] or the social cognitive theory (SCT) alone [33,37] or in combination with other theories such as the reinforcement theory of motivation [48], the multiple intelligences theory [35], or the protection motivation theory [34]. The serious games with a focus on treatment used theories, theoretical constructs, or intervention techniques for behavior change, such as the I-Change Model [41], coping skill training [39], cognitive bias modification of attention [49], cognitive behavioral therapy [27,45], or cue exposure therapy [40]. A total of 24% (7/29) of the studies, all with prevention objectives, did not mention what theory, theoretical construct, or intervention technique was implemented in the serious game. Table 2 presents in detail the theoretical basis reported in each study split into theory and theoretical construct or intervention technique.

**Table 2:** Summary of the main characteristics of the reviewed serious games (N=29).

Study and game name	Substance addressed	Type of intervention	Theoretical basis		Computational techniques	Evaluation protocol	Mechanism for data security and confidentiality	Main reported results
			Theory	Theoretical construct or intervention technique				
Cheng et al [31]—Drugs and the Brain	Methamphetamine	Prevention—education on the impact of methamphetamine abuse on the brain	Not specified	Not specified	Virtual reality learning environment	Pilot study (pre- and posttest measurements) with 175 visitors (aged 6 to 82 years) to a museum	Not specified	Improvement of knowledge of basic neuroscience concepts and understanding and attitudes toward the impact of methamphetamine abuse.
Klisch et al [30]—Uncommon Scents	Inhalants (toxic chemicals)	Prevention—education on the biological consequences and risk of inhaling toxic chemicals	Not specified	Constructive learning	Web-based serious game	Pilot study (pre- and posttest measurements) involving 444 middle school students (sixth-, seventh-, and eighth-graders)	Data collected anonymously	Significant gains in science content knowledge were obtained. A shift to more negative attitudes toward inhalants was also observed. The most negative shift was among eighth-grade students. Posttest knowledge gains were the strongest predictor of attitude change across all grade levels.
Klisch et al [29]—Bitter Pill and Fatal Interactions	Prescription drugs	Prevention—education on the risk of prescription drug abuse	Theory of reasoned action	Not specified	Web-based serious game	Pilot study (pre- and posttest measurements) with 179 high school students (11th and 12th grade) divided into 2 groups assigning each group to a different game (Bitter Pill and Fatal Interactions)	Data collected anonymously	In both groups, the attitudes toward prescription drug abuse became significantly more negative. A moderate effect size in the Bitter Pill group and a large effect size in the Fatal Interactions group were found.
Sánchez and Bartel [39]—Arise	Alcohol and drugs	Treatment—education and building of coping skills for relapse prevention	Not specified	Coping skill training	Web-based serious game	Feasibility study (posttest measurements) with 8 treatment providers and a pilot study (posttest measurements) with 9 adolescents in substance abuse treatment	Data collected anonymously	The treatment providers rated the serious game with high likability and usefulness. Adolescents rated the game as easy to use, highly acceptable, and very useful as a substance abuse relapse prevention tool.
Elias-Lambert et al [28]—Choices & Consequences	Not explicitly mentioned; the study only refers to “substance abuse” and relationship violence	Prevention—education on the substance abuse and relationship violence challenges, the possible actions, and the consequences associated with those actions	Not specified	Situated experiential learning	Multiuser, mobile-based serious game	Exploratory proof of concept (posttest measurements; 6 focus groups) with 44 youth school students (aged >14 years)	Not specified	Participants reported enjoyment related to the visual and auditory aspects of the game and the facts provided to give further information about selected actions and their consequences. In terms of engagement, some of the participants reported that the scenarios were realistic and could connect and engage with them. Other feedback indicates game preference as a learning tool over traditional formats.
Jander et al [41]—What happened?	Alcohol	Treatment—promotes behavior change through developing a favorable attitude, experiencing positive social influences, and developing high self-efficacy toward the behavior	The I-Change Model	Social norms, perceived pressure, and self-efficacy	Web-based serious game	Cluster RCT^a^ (pre- and posttest measurements)—34 schools with 2649 adolescents (aged between 15 and 19 years) divided into experimental (1622) and control (1027) groups	Not specified	The serious game was effective in reducing binge drinking in adolescents aged 15 and 16 years when they participated in at least two intervention sessions. Interaction effects were found between excessive drinking and educational level and between weekly consumption and age. Additional analyses revealed that prolonged use of the intervention was associated with stronger effects for binge drinking. However, overall adherence to the intervention was low.
Epstein et al [32]—Bacon Brains	Alcohol and drugs	Prevention—education on the science of addiction and how alcohol and other drugs affect the brain	Not specified	Not specified	Web-based serious game	RCT (pre- and posttest measurements) with 244 students (sixth to eighth grade) aged 11 to 15 years	Not specified	A more significant knowledge gain among the intervention groups was found compared with the control group. The intervention was helpful in teaching students core concepts about the science of addiction, and the knowledge they learned persisted through posttest assessment. Girls acquired knowledge gains in the collaborative and competitive game conditions, whereas boys demonstrated similar gains only in the competitive condition. Students in the experimental conditions reported enjoying playing the games more than students in the control condition.
Hookham et al [26,27]—Shadow	Alcohol (comorbidity with depression)	Treatment—promotion of behavioral and cognitive change related to mood and misuse of alcohol	Not specified	Cognitive behavioral therapy and motivational interviewing	Web-based serious game where the storyline is based on branching of predefined dialogues	Comparative study (posttest measurements, within-subject) between a gamified and nongamified version of a web-based alcohol abuse and depression treatment with 10 university students (aged 18-30 years)	Not specified	No significant differences were found between the gamified and nongamified versions of the web-based treatment in terms of usability, ease of use, perceived usefulness, or engagement.
Hughes et al [36]—CollegeLiVE	Alcohol and drugs	Prevention—presents typical situations to practice social skills, protective behaviors, and self-reflection	Social cognitive theory and interactive performance theory	Not specified	Virtual reality scenario based on a computational framework that facilitates the creation and remote control of avatars for interaction purposes	Quasi-experimental study (pre- and posttest measurements) with 68 university students (45 in the intervention group and 23 in the control group) aged ≥18 years	Not specified	No significant differences were found between the intervention and control groups when questioned about specific protective behaviors. Nevertheless, the participants in the intervention group were significantly more likely to implement protective behaviors such as trying to stop somebody they knew from deciding to drive after drinking and using a designated driver than the participants in the control group.
Gamberini et al [50]—no name provided	Alcohol and drugs	Prevention—education on and awareness of the risks related to the consumption of psychoactive substances for partygoers	Not specified	Not specified	Multiplayer web-based serious game	Two pilot studies (pre- and posttest measurements), one to assess risk awareness and user experience with 227 participants (mean age 24.7, SD 6.78 years) and the other to assess knowledge increase with 81 participants (mean age 23.4, SD 2.02 years)	Data collected anonymously	The results from the first pilot study indicate that the user experience was high. In the second pilot study, significant differences were found in risk assessment and knowledge of substance consumption risks and coping skills between pre- and postgame sessions.
Boendermaker et al [49]—Shots	Alcohol	Treatment—cognitive retraining of selective attention toward alcohol	Not specified	CBM-A^b^	Not specified	RCT (pre- and posttest measurements) with 96 heavy-drinking university students (aged 18-28 years). A total of 33 participants were assigned to the gamified version of CBM-A, 30 were assigned to a regular version of CBM-A, and 33 were assigned to a placebo version.	Not specified	There was an overall decline in alcohol attentional bias, but this effect was primarily driven by the regular training condition. Motivation to train decreased equally in all conditions, indicating that the motivational elements in the gamified version could not sufficiently counteract the tiresome nature of the training. Moreover, motivation to change with respect to planning to drink less in the future increased in the regular and placebo training but decreased in the gamified training condition, which may indicate potential detrimental effects of disappointing gamification.
Boyle et al [51]—CampusGANDR	Alcohol	Treatment—modification of behavior by correcting normative perceptions and inducing reductions in alcohol use	Not specified	Personalized normative feedback	Web-based serious game with Facebook log-in credentials to simulate a social game (but no real social connection)	RCT (pre- and posttest measurements and follow-up) with 237 undergraduate students aged between 18 and 24 years. A total of 113 students were assigned to the use of the serious game, and 124 were assigned to the standard intervention (control group).	Not specified	Participants in the serious game condition reported significantly reduced peer drinking norms and alcohol consumption at the 2-week follow-up compared with participants who received the standard intervention. A mediation model demonstrated that this effect was driven by larger reductions in perceived drinking norms among participants assigned to the serious game.
Damasceno et al [37]—no name provided	Drugs	Prevention—education on the damage of drug abuse and submission of the player to moral decisions	Social cognitive theory	Not specified	PC-based serious game	Pilot study (posttest measurements) with 69 school students (mean age 13.7 years)	Not specified	On the basis of the collection and analysis of in-game behaviors (statistics such as the time spent, correct and wrong answers, and response time), the authors present a general picture of the differences in game performance among groups of users. The authors conclude that serious games could be a powerful tool for drug abuse prevention as an instructional mechanism and for the identification of risk behaviors.
Stapinski et al [52]—Pure Rush	Drugs (focused on cannabis, ecstasy, methamphetamine, and hallucinogens)	Prevention—education on the effects associated with each drug and pairing drug-related cues with negative stimuli	Not specified	Not specified	Web-based serious game with a mobile version for Android devices	Feasibility study (posttest measurements) with 25 students aged between 14 and 17 years and an RCT (pre- and posttest measurements) in 9 schools with 281 students aged between 13 and 16 years. A total of 148 students were assigned to game sessions, and 133 were assigned to the control group.	Data collected anonymously	Results from the feasibility study indicate that students enjoyed playing Pure Rush and found the game age-appropriate and the game’s infographics understandable and appealing. Results from the RCT indicate significant knowledge increase from before to after the intervention in both groups (no significant difference between groups). Knowledge gain was greater in the game condition than in the control group but only in female students. Very low intentions to use illicit drugs were observed in both groups. No significant differences were found in lesson engagement and in future intentions to use drugs between the intervention and control groups.
Earle et al [42]—CampusGANDRV2	Alcohol	Treatment—modification of behavior by correcting normative perceptions and inducing reductions in alcohol use	Self-determination theory	Personalized normative feedback	Mobile-based serious game	RCT (pre- and posttest measurements and follow-up) with 276 first-year university students. A total of 93 students were assigned to the game session receiving standard feedback, 90 were assigned to the game plus supplemented feedback on their perceptions and behaviors, and 93 were assigned to a control group.	Not specified	Participants who were assigned to the game with the supplemented feedback reduced their drinking significantly during the 2 months after the intervention in comparison with control participants. Reduction in drinking behavior was stronger among heavy drinkers.
Duncan et al [53]—SmokeSCREEN	Drugs	Prevention—education and presentation of social situations to develop behavioral skills associated with primary prevention of cigarette and marijuana use	Not specified	Not specified	Mobile-based serious game	Pilot study (pre- and posttest measurements) with 25 adolescents aged between 11 and 14 years	Not specified	Improvements in knowledge were found for both cigarette and marijuana between baseline and after playing. No significant changes in perceived social norms toward both substances were reported. The players provided positive feedback about their experience with the serious game.
Metcalf et al [45]—Take Control	Alcohol and cigarette	Treatment—practice of refusal skills and increase in self-efficacy by denying trigger or cue items in a nonthreatening environment	Not specified	Cue exposure therapy, extinction therapy, virtual reality therapy, and cognitive behavioral therapy	PC-based serious game with Kinect	Quasi-experimental wait-list study (pre- and posttest measurements) with 61 participants aged >18 years. A total of 29 participants were assigned to the intervention group, and 32 were assigned to the wait-list control group.	Data collected anonymously	The results reported that substance use decreased or remained for most users, although more for alcohol consumers than for tobacco users. Participants in recovery for alcohol use reported more benefit than those in recovery for tobacco use, with a statistically significant increase in self-efficacy, attitude, and intended behavior. Participants found the game engaging and fun and felt that playing it would support recovery efforts.
Kapitány-Fövény et al [46]—Once Upon a High	Alcohol and drugs	Prevention—education on the epidemiology and risks of substance use, promotion of health-conscious behavior, and decrease in stigma and negative attitudes and increase in willingness to help peers with substance use problems	Not specified	Self-efficacy	Mobile-based app containing 2 serious games	Quasi-experimental study (pre- and posttest measurements) with 386 students aged between 14 and 18 years from 4 different schools. A total of 255 students were assigned to the intervention group (app use), and 131 were assigned to the control (nonapp use) group.	Data collected anonymously	Users in the intervention group showed a greater decrease in energy drink consumption. No relevant differences between the 2 groups were found in knowledge gaining on psychoactive substances and physical exercise frequency. A correlation between the app’s perceived usefulness and a decreasing frequency of past-month alcohol use was found. Results also indicate that a decrease in negative attitudes toward substance users might be a risk factor for increasing past-month alcohol consumption.
Gamberini et al [54]—no name provided	Alcohol and drugs	Prevention—education on the potential risks of psychoactive substance abuse during nightlife events	Not specified	Not specified	Multiplayer web-based serious game	Quasi-experimental comparative study (posttest measurements) with 136 young adults (mean age 23.5 years). A total of 67 participants used the game, and 69 read leaflets with information on the potential risks of psychoactive substance abuse.	Data collected anonymously	The evaluations of the game credibility and effectiveness were positive. No significant difference was found between game and leaflets except for informativeness, on which leaflets performed better than the game.
Abroms et al [48]—Recovery Warrior 2.0	Drugs	Treatment—helping the patients in the development of negative associations with drugs and acquisition of drug refusal skills	Social cognitive theory and reinforcement theory of motivation	Repetition priming	PC-based serious game with Kinect for body motion and voice recognition	RCT (pre- and posttest measurements and follow-up) with 80 participants aged between 15 and 25 years under a drug treatment program. A total of 36 participants were assigned to the use of the game+treatment as usual, and 44 were assigned to the control group (treatment as usual only).	Not specified	Participants in the intervention group mostly agreed that they would use the refusal skills taught by the game and reported attending more outpatient counseling sessions than those in the control group, but the difference was not significant. Cravings declined for both groups from baseline to the 4- and 8-week follow-up, but the differences between groups were not statistically significant. Self-efficacy fluctuated slightly but did not change widely between the intervention and control groups. The game had no effect on drug use at 4 or 8 weeks after discharge, with the exception of a benefit reported at the 4-week follow-up among participants receiving treatment for marijuana addiction.
Carvalho et al [38]—JiB	Alcohol	Prevention—education on the consequences of alcohol abuse promoting empowerment of the individual, traditional cultures, and social responsibility	Not specified	Not specified	Mobile-based serious game	Pilot study (pre- and posttest measurements) with 23 participants aged between 20 and 29 years	Data collected anonymously	The results were analyzed by dividing the participants into habitual and nonhabitual players. The game presented more positive than negative effects on all users. For habitual players, the game did not have a high level of challenge owing to the low difficulty and small learning curve. A higher game tension was found in nonhabitual players versus habitual players.
Willmott et al [47]—Perfect Pour and Dumb Driver	Alcohol	Prevention—education on the physiological effects of varying levels of blood alcohol concentration on driving ability (Dumb Driver) and on the standard alcohol content of 6 types of alcoholic drinks through a multilevel test of pouring accuracy (Perfect Pour)	Theory of reasoned action	Subjective norms and attitudes	Web-based serious game	Pilot study (pre- and posttest measurements) with 303 students aged between 12 and 17 years	Not specified	A positive relationship between average game duration and attitude was found, indicating that the longer the participants played Perfect Pour, the more positive their attitudes became toward binge drinking. On the contrary, a negative relationship between average score and attitudes indicates that the higher the players scored in Perfect Pour, the more negative their attitudes became toward binge drinking. No significant associations were observed among gameplay metrics, attitudes, and subjective norms for Dumb Driver.
Tan et al [55]—Drug Defense	Alcohol	Prevention—education on the consequences of excessive alcohol use	Social cognitive theory and multiple intelligences theory	Not specified	Mobile-based serious game	Pilot test (pre- and posttest measurements) with 69 university students (aged 18 to 21 years)	Not mentioned	Significant differences between pre- and posttest measurements were found, indicating an increase in knowledge of alcohol. Playability was good according to the values obtained in the evaluation of gameplay, story, and mechanics of the game.
Mostajeran et al [40]—no name provided	Alcohol	Treatment—minimize the occurrence of relapse by practicing to avoid alcohol in a simulated supermarket	Self-determination theory	Cue exposure therapy and approach-avoidance training	PC-based serious game with virtual reality using a head-mounted display	Comparative study (posttest measurements, within-subject) with 13 participants (aged 22 to 35 years). All the participants were assigned to a gamified version of the approach-avoidance training and cue exposure therapy and a nongamified version of the approach-avoidance training.	Not mentioned	All the gamified versions received high usability scores. The gamified version of the approach-avoidance training was more cognitively demanding than the nongamified version. The gamified version of the approach-avoidance training was more motivating than the nongamified version. Participants made fewer errors in the gamified version of the approach-avoidance training than in the nongamified version. The users preferred the mini-game of the approach-avoidance training than the mini-game of the cue exposure therapy.
Yap et al [35]—Drug Defense	Alcohol	Prevention—education on the consequences of excessive alcohol use	Social cognitive theory and multiple intelligences theory	Not specified	Mobile-based serious game	RCT (pre- and posttest measurements) with 140 university students (aged 18 to 21 years). A total of 69 were assigned to the use of the mobile-based game, and 71 were assigned to a video intervention (control group).	Encoding of data and password protection	Participants who used the game showed a significant increase in knowledge scores and a decrease in intent to use but not in actual use. Participants in the video intervention reported a significant increase in knowledge and a decrease in both intent and use. Findings also showed a significant difference in alcohol knowledge for both game and video settings, with the game having a larger effect size than the video.
Ozer et al [33]—INSPIRE	Alcohol	Prevention—education on personal efficacy and skills by providing opportunities to practice strategies for reducing risky health behaviors	Social cognitive theory	Self-efficacy and self-regulation	PC-based serious game with interactive narrative experiences	Pilot study (posttest measurements) with 20 adolescents (aged 14 to 19 years)	Not specified	Trace-log data and self-report questionnaires indicate that participants found the game to be engaging, believable, and relevant to their lives. Within the game, participants also successfully used a range of strategies for avoiding alcohol use. The participants accessed fewer in-game objects than anticipated and spent less time examining the associated infographics designed to affect knowledge of the effects of alcohol use.
Hong et al [34]—One Shot	Alcohol	Prevention—education on and practice of drinking refusal self-efficacy	Social cognitive theory and protection motivation theory	Not specified	Web-based serious game	Quasi-experimental study (1-group, pre- and posttest measurements) with 550 young adults (aged 21 to 25 years) at risk of binge drinking	Not specified	Results show improvements from before to after the game in intention to drink less and in 5 of the 7 indicators of drinking refusal self-efficacy. Risky alcohol decisions within the game and game time predicted enjoyment, which, in turn, predicted intention to drink less and drinking refusal self-efficacy. Enjoyment significantly mediated the effects of game time and risky alcohol decisions on intention to drink less and drinking refusal self-efficacy.
Boyle et al [43]—GANDR	Alcohol	Treatment—modification of behavior by correcting normative perceptions and inducing reductions in alcohol use	Self-determination theory	Personalized normative feedback	Web application (multi-platform) serious game	RCT (pretest measurements and follow-up 1 month later) with 223 first-year university alcohol-experienced students (mean age 18.05 years). A total of 74 students were assigned to the gamified-only intervention, 74 were assigned to the gamified social media intervention, and 75 were assigned to the nongamified intervention (control group).	Not specified	Reported results indicate a significantly greater reduction in drinking at follow-up in the gamified social media condition than in the traditional (nongamified) condition. It was unclear whether the gamified-only condition would lead to greater reductions in drinking than the traditional condition. The gamified-only condition did not lead to a significantly greater reduction in drinking at follow-up compared with the nongamified condition. Relative to the nongamified condition, the gamified social media condition led to significantly greater reductions in drinking for those who were lighter drinkers with less exposure to alcohol-related content on social media.
Boyle et al [44]—LezParlay	Alcohol	Treatment**—**reduction of risks related to alcohol consumption	Self-determination theory	Personalized normative feedback	Web application (multi-platform) serious game	RCT (pretest measurements and follow-up at 2 and 4 months) with 499 LBQ^c^ women aged 21 to >40 years with moderate and heavy alcohol consumption. A total of 143 women were assigned to the gamified intervention on alcohol use and stigma coping, 179 were assigned to the gamified intervention on alcohol use only, and 177 were assigned to the control group.	Not specified	Obtained results indicate that participants who received the intervention on alcohol use and both alcohol use and stigma coping had similar reductions in their weekly drinks, peak drinks, and negative consequences relative to those in the control group at the 2-month follow-up. However, at the 4-month follow-up, reductions in alcohol consumption outcomes faded among those who received the alcohol-only intervention, whereas they remained relatively robust among those who received both the alcohol use and coping intervention. Regarding feasibility, the participants reported the competition to be highly acceptable and psychologically beneficial as a whole.

^a^RCT: randomized controlled trial.

^b^CBM-A: cognitive bias modification of attention.

^c^LBQ: lesbian, bisexual, and queer.

### Computer-Based Techniques Used

In most analyzed studies (24/29, 83%), little technical detail was provided about the computer-based methods or techniques used to develop the serious games. Most of the studies only mentioned the platform where the serious game could be executed: web-based (11/29, 38%), mobile app (7/29, 24%), multi-platform (3/29, 10%), or PC-based (2/29, 7%). The studies that provided more technical detail mentioned the use of virtual reality for the creation of immersive environments [31,40], the use of a Kinect device for a motion-based interaction [45,48], the implementation of branching predefined dialogues to create interactive narrative experiences [26,27,33], the integration with Facebook to simulate a social serious game [51], and the use of a computational framework [56] that facilitated the creation and remote control (as in Wizard-of-Oz scenarios) of avatars for interaction purposes [36].

### Data Security and Confidentiality

Most of the reviewed studies (19/29, 66%) did not explicitly mention whether a specific mechanism was implemented in the serious game to assure the data protection and confidentiality of the users. Although all the studies (29/29, 100%) reported that an institutional ethical committee had approved the protocol implemented to evaluate the serious game, only some (9/29, 31%) mentioned the type of data collected from the participants and how confidentiality was guaranteed. The most frequent type of data collected in these studies was sociodemographic information (eg, sex, age, and ethnicity) without collecting identifiable information, maintaining the anonymity of the participants [29,30,38,39,45,46,50,52,54]. Only 3% (1/29) of the studies mentioned encoding of the data and password protection [35].

### Evaluation Procedures and Reported Results

The analyzed studies reported the evaluation of the serious games through RCTs (10/29, 34%), quasi-experimental studies (5/29, 17%), pilot studies (11/29, 38%), comparative studies (2/29, 7%), and a proof-of-concept study (1/29, 3%). The reported results from the RCTs were in general positive, describing significant differences between the intervention and control groups in terms of decreasing binge drinking [41], reducing peer drinking norms and alcohol use [42-44,51], gaining knowledge on how alcohol and drugs affect the brain [32], increasing substance-related knowledge (but not decreasing psychoactive substance use) [46], being more accepting toward using refusal skills of drug use (but no effect was found on drug use) [48], or increasing knowledge of the effects of alcohol use and protective behavioral techniques and decreasing intention of alcohol use (but not actual use) [35].

Nevertheless, some of these RCT studies reported mixed results in the use of serious games: the longer the average game duration, the more positive the attitude toward binge drinking that was found. However, the same study reported that the higher the players scored in the game, the more negative their attitudes became toward binge drinking [47]. Similarly, no significant differences were found in learning outcomes on drug education (except for women, where knowledge gaining was greater in the intervention than in the control group), lesson engagement, or future intentions to use drugs between the intervention and control groups [52]. Moreover, the results reported in the study by Boendermaker et al [49] indicate that a gamified version of a cognitive bias modification of attention program reduced the motivation to train, and self-reported drinking behavior was not affected.

In addition, the results reported from quasi-experimental studies reflected positive outcomes after the use of serious games. The participants reported in the study by Klisch et al [29] demonstrated an increase in negative attitudes toward prescription drug abuse after the use of serious games, and the games’ ratings for satisfaction and engagement were above average. Alcohol use decreased in individuals under substance use treatment during an intervention using a serious game, and there was a significant increase in self-efficacy, attitude, and behavior to prevent relapse. Nevertheless, these positive results decreased over time after the last playing session [45]. The study by Hong et al [34] reported that participants improved from before to after the game in intention to drink less alcohol and in drinking refusal self-efficacy, where the enjoyment of the game was a key feature. The results reported in the study by Hughes et al [36] indicate that participants from the intervention group were more likely to adopt protective behaviors such as trying to stop somebody from driving after drinking and using a designated driver than participants in the control condition. One of these studies also demonstrated that participants in a game session to provide information about potential risks of substance abuse during nightlife events evaluated the serious game to be as positive and credible as more traditional information tools such as leaflets [54].

The rest of the papers (11/29, 38%) reported pilot studies where the developed serious games were assessed with a group of participants in terms of usability and acceptability, including outcomes such as perceived utility, engagement, enjoyment, or game experience [33,38,39]; the performance of the players to assess the suitability of serious games as a learning tool for the prevention of drug consumption [37]; and the effectivity in terms of change in knowledge and attitudes toward substance use [30,31,50,53]. Finally, the 7% (2/29) of comparative studies assessed the differences between a gamified and a nongamified version of existent treatment programs. One of these studies did not find relevant differences in usability, ease of use, and perceived usefulness between the 2 treatment versions [26,27]. The other comparative study reported differences in motivation, cognitive demand, and the number of errors made between the 2 versions. The gamified version was more motivating and cognitively demanding, and the participants made fewer mistakes [40]. Table 2 summarizes the main extracted data from each analyzed study.

## Discussion

### Principal Findings

Given the increasing interest in developing serious games as digital interventions to support the prevention and treatment of alcohol and drug consumption, it is relevant for future works in the area to identify key features that designers of serious games should consider. This scoping review contributes by identifying and summarizing the description of 7 main characteristics that reflect how these games are currently designed and what are the intervention results reported. The increase in studies focused on developing and evaluating this type of serious game is evident in this review. Previous similar reviews (although considering only serious games with learning purposes) published in 2014 [18] and 2016 [19] found 12 and 8 serious games, respectively.

By contrast, our review found 29 serious games considering only the previous 12 years (2010-2022), with most (24/29, 83%) published in the last 6 years, from 2016 onward. The main substance addressed in the serious games included in this review was alcohol by itself or with other drugs. This result is not surprising as alcohol, a licit substance, is still the most consumed by the young population worldwide, and it causes more direct and indirect deaths than illicit drugs [57]. As alcohol and drugs share many risk behaviors and health consequences, serious game scenarios and narratives can be applied to both types of substances. Nevertheless, it is necessary to carry out more specific studies to assess if any positive effects from serious games could be applied equally to alcohol and illicit drugs without changing much of the game’s mechanics.

Two-thirds of the 29 analyzed serious games (18/29, 62%) focused on supporting preventive interventions, whereas the rest (11/29, 38%) were developed to complement the treatment process. This disparity could be because preventive serious games are mainly developed to support educative aspects associated with, for example, risk behaviors, protective skills, and the negative consequences of alcohol and drug abuse. As learning material, these serious games do not necessarily require the direct or close supervision of a specialist and have the potential to reach a high number of the intended population (as part of formal curricula or not). By contrast, the serious games focused on treatment must be carefully designed considering the characteristics of potential users with high consumption levels or addiction problems. Moreover, to maximize the effectiveness and security of using these serious games, the treatment provider must necessarily be involved in the stages of the treatment addressed by the game. In this sense, serious games are not as different from other digital-based tools where their benefits to the treatment process are higher if used under specialist guidance or supervision [58,59].

Using a background theory in any intervention, including serious game–based interventions, is essential to understand not only what interventions work but why they work [60]. In this sense, it is noticeable that most of the reviewed studies (22/29, 76%) mentioned the theoretical basis (theory, theoretical constructs, or intervention techniques or all three) used in the serious games. The theoretical background of the reviewed serious games also depended on their prevention or treatment support objectives. For the games used with prevention objectives, different theories were used to design the games’ mechanics and contents, highlighting the SCT. The main posits of the SCT (formerly known as social learning theory) consider that the acquisition and maintenance of specific (healthy or not healthy) behaviors depend on reciprocal interaction between the individual and their social context mediated by positive and negative reinforcements [61]. Thus, this theory offers promising opportunities to represent this interaction through different game scenes where the player can observe (and learn) the consequences of their own or others’ actions (vicarious learning) in realistic scenarios associated with substance use.

It is noteworthy that 39% (7/18) of the analyzed serious games with prevention objectives (through education) did not explicitly mention any theoretical background. This lack of specification would make it difficult to further analyze what specific game mechanics (and the theoretical tenets on which they are based) positively affect the learning process to prevent substance use. By contrast, the serious games developed to support the treatment process (11/29, 38%) were based on different intervention techniques, with none standing out from the others. Depending on the desired outcomes, different theoretical constructs and intervention techniques were used, including cognitive behavioral therapy and motivational interviewing to promote change of behaviors related to substance abuse or cognitive bias modification for cognitive retraining of selective attention toward substances, to name a few. Some others (5/29, 17%) took advantage of the user interface’s characteristics to implement, for example, cue exposure therapy and approach-avoidance training in virtual reality scenarios accessed through Kinect or head-mounted displays.

Regarding the computational techniques and approaches used, most of the included serious games (23/29, 79%) only specified the platform where the game could be deployed: web, mobile, or stand-alone environments. Some of them also specified the implementation of multiplayer or social network characteristics and virtual reality scenarios. Nevertheless, none of the reviewed serious games implemented more advanced techniques and interfaces such as brain-computer interaction and eye and head tracking combined with machine learning algorithms to, for example, automatically detect individual characteristics (eg, mood, emotions, and attention) of the players and personalize or adapt the game contents and mechanics. These techniques are currently used in serious games with evident advantages in other contexts, particularly for learning purposes [62-64]. Thus, a future direction would be to develop and evaluate serious games implementing these computational approaches and assess whether their use improves the desired outcomes in preventing and treating substance use.

The use of serious games for health raises particular challenges as their objective is not only to entertain but also to influence users’ attitudes and behaviors that would affect their everyday life [65]. One of these challenges is to decide what data are collected through the serious game, how this information is used, and how to guarantee user data security and confidentiality. It is noticeable that only 34% (10/29) of the included studies mentioned issues related to privacy. This result, of course, does not mean that the rest of the reviewed serious games did not implement a data security and confidential mechanism as all the reviewed works (29/29, 100%) reported the approval of their use in studies involving humans by an ethical board. A protocol for ethical approval in digital interventions usually must define what data are collected, where they are stored, who will use them, and how privacy and confidentiality are guaranteed [66]. Nevertheless, it seems necessary that this information be included when reporting the design, implementation, and evaluation of serious games for health to identify how the challenge of data security and privacy is addressed instead of only mentioning that the data are collected anonymously.

Another of the relevant current and future challenges in developing these types of serious games is to evaluate their actual efficacy on the addressed population. The assessment of evaluation results is particularly relevant for the effective adoption and appropriation of serious games (as digital technology for health), where evidence of the contributions of digital health interventions to the performance of health systems and their impact on people’s health and well-being is one of the key activities of the WHO global strategy on digital health [67]. Many of the reviewed papers (11/29, 38%) reported feasibility and pilot studies focused on, for example, usability, engagement, game experience, or perceived utility of the serious games. There were a few quasi-experimental studies (5/29, 17%) where the main factors assessed were changes in intentions or attitudes toward substance consumption and knowledge gaining. Overall, the reported results from these quasi-experimental studies were positive considering the observed differences in the participants at pre- and posttest measurements. Still, all these studies warrant a follow-up assessment to know whether these observed effects are maintained in the long run.

Similarly, the reported RCTs also highlighted some positive effects when comparing the intervention and control groups in terms of reducing substance use, increasing knowledge, or being more accepting of refusal skills. Nevertheless, other RCTs (3/10, 30%) did not find significant differences, and 10% (1/10) even warned about gamification’s detrimental effects [49]. A follow-up assessment was included in 50% (5/10) of the reported RCTs, assessing the long-term effect after 2 months of the intervention in 60% (3/5) of them. One of these studies reported that cravings declined from baseline to the 4- and 8-week follow-up, although differences between the intervention and control groups were not significant [48]. Another study reported that, after 4 months, reductions in alcohol consumption faded in the group of participants that received the alcohol-only intervention and remained *relatively* robust in the group that received the alcohol and coping stigma intervention [44]. Thus, more RCT studies are necessary focusing on identifying relevant outcomes such as changes in attitudes, motivation, and behaviors. Moreover, including follow-up assessments for more prolonged periods and the reproducibility of these studies would help understand better the effect of serious game–based interventions for both prevention and treatment of substance use.

### Limitations

This study has some limitations related to the selection procedure. One of these limitations is that only studies reporting an evaluation procedure were included. Thus, some serious games that implemented more sophisticated computational techniques, such as machine learning, other artificial intelligence methods, or other interactive devices that had not yet been evaluated, were not considered. Moreover, the only sources used to identify serious games were academic repositories. No other sources such as app stores were considered to assess whether some of these available apps included gamification techniques aimed at preventing or supporting substance use treatment. Nevertheless, the contribution of this study is relevant as it highlights the current state of the art in the research and development of this type of serious games, identifying relevant characteristics such as the substance addressed, their theoretical roots, the computational techniques used, the mechanism for data security and confidentiality, and the results obtained from an evaluation protocol.

### Conclusions

The development and evaluation of serious games to support prevention and treatment interventions for substance use have increased in the last decade. The review presented in this paper describes a set of main characteristics of 29 serious games evaluated with adolescents and young adults aiming to prevent and reduce the consumption of alcohol and drugs. Most of the analyzed serious games were designed to prevent or reduce alcohol consumption. Those developed with prevention objectives were also the majority compared with those developed to support treatment. The reported evaluation protocols included mostly pilot studies, quasi-experimental studies, and RCTs.

Overall, most of the reviewed serious games reported positive results regarding acceptability, usability, increased knowledge, and change in attitudes and behaviors toward alcohol and drug consumption. An area of future research is the incorporation of other human-computer interaction and artificial intelligence techniques to identify relevant user data, allowing for the personalization of the offered interventions. In addition, more studies—that facilitate reproducibility—are necessary to better identify the long-term effects of these serious games (and whether boosted playing sessions are necessary after some time) and what specific game mechanics are more useful for preventive or treatment interventions.

## Abbreviations

PRISMA-ScR: Preferred Reporting Items for Systematic Reviews and Meta-Analyses extension for Scoping Reviews
RCT: randomized controlled trial
SCT: social cognitive theory
WHO: World Health Organization
